# Antimicrobial Usage and Presence of Extended‐Spectrum *β*‐Lactamase–Producing Enterobacterales in Healthy Chickens From Farms in Grand‐Abidjan and Korhogo, Côte d′Ivoire

**DOI:** 10.1155/ijm/7418124

**Published:** 2026-10-21

**Authors:** Christiane You Essoh, Gilles Alex Pakora, Daouda Toure, Inès Cinthia A. Yebouet, Bérenger Aristide A. Ako, Jemima Arlette Ayebie, Naomie Dali, Sinon-Pierre Assanvo N′guetta, Adèle Kakou-N′douba

**Affiliations:** ^1^ Laboratory of Biotechnology and Agro-Food Valorization UFR of Biological Sciences Universite Peleforo Gon Coulibaly Korhogo Ivory Coast; ^2^ Laboratory of Biology and Health UFR of Biosciences Universite Felix Houphouet-Boigny Abidjan Ivory Coast; ^3^ Laboratory of Microbiology Biotechnology and Bioinformatics Institut National Polytechnique Felix Houphouet-Boigny Yamoussoukro Ivory Coast; ^4^ Department of Parasitology-Mycology Institut Pasteur de Cote d′Ivoire Abidjan Ivory Coast; ^5^ Doctoral School of Biology-Environment-Health UFR of Biosciences Universite Felix Houphouet-Boigny Abidjan Ivory Coast; ^6^ Laboratory of Biotechnology Agriculture and Biological Resources Valorization UFR of Biosciences Universite Felix Houphouet-Boigny Abidjan Ivory Coast; ^7^ Laboratory of Bacteriology-Virology UFR of Medical Sciences Universite Felix Houphouet-Boigny Abidjan Ivory Coast

**Keywords:** Côte d′Ivoire, Enterobacterales, ESBL genes, poultry farms

## Abstract

**Background:**

The global spread of extended‐spectrum *β*‐lactamase (ESBL)–producing Enterobacterales in livestock poses critical threats to human and animal health, particularly in low‐ and middle‐income countries. This study investigated the prevalence, antibiotic resistance profiles, and genetic determinants of ESBL‐producing Enterobacterales in healthy chickens from poultry farms in Grand‐Abidjan and Korhogo, Côte d′Ivoire, alongside antibiotic use practices in the sector.

**Methods:**

A semistructured questionnaire assessed veterinary practices across 26 farms, whereas chicken fecal samples from 27 farms were screened for third‐generation cephalosporin‐resistant Enterobacterales. Phenotypic and genotypic characterization of ESBL producers was performed, along with antimicrobial susceptibility testing against 12 antibiotics.

**Results:**

All farmers reported antibiotic use, primarily tetracyclines (76.9%), macrolides (69.2%), polymyxins (61.5%), aminoglycosides (57.7%), and sulfonamides (53.8%), mainly for disease prophylaxis (65.38%) and often without veterinary supervision. ESBL‐producing Enterobacterales were detected in 96.3% (26/27) of fecal samples, yielding 90 isolates identified as clinically relevant species: *Klebsiella pneumoniae* (42.2%, 38/90), *Escherichia coli* (38.9%, 35/90), and *Enterobacter* spp. (18.9%, 17/90). Overall, 74.8% (63/90) of isolates were resistant to trimethoprim–sulfamethoxazole and 57.8% to amoxicillin–clavulanic acid, whereas no resistance to carbapenems was observed. Molecular characterization revealed the presence of *bla*_CTX-M_, *bla*_SHV_, and *bla*_TEM_ genes, with *bla*_CTX-M_ being the most prevalent (77.8%, 70/90), followed by *bla*_SHV_ (54.4%, 49/90) and *bla*_TEM_ (31.1%, 28/90). These genes were coexpressed in half of all isolates, predominantly in *K. pneumoniae* and *Enterobacter* spp.

**Conclusion:**

This study highlights the widespread dissemination of ESBL‐producing Enterobacterales in poultry farms in Côte d′Ivoire, emphasizing the role of poultry as a reservoir for resistant bacteria and underscoring the urgent need for integrated surveillance and antimicrobial stewardship within a One Health framework.

## 1. Introduction

Antimicrobial resistance (AMR) is one of the most critical global health threats of the 21st century, directly undermining the efficacy of life‐saving antibiotics [1, 2]. According to a recent World Health Organization (WHO) report, global surveillance data reveal that resistance rates increased by over 40% between 2018 and 2023 [3]. Without decisive intervention, AMR could escalate by 70% by 2050, potentially causing up to 10 million annual deaths [1, 4]. The misuse and overuse of antibiotics in livestock production is a major driver of AMR, accelerating the evolution and global spread of resistant bacteria [5]. Furthermore, low‐ and middle‐income countries, particularly in sub‐Saharan Africa, bear a disproportionate burden due to fragile healthcare systems, socioeconomic disparities, weak pharmaceutical regulation, poor sanitation, and limited surveillance capacity [6]. These factors are the primary drivers of AMR in the region, which could face 4.1 million AMR‐related deaths annually by 2050 [4, 6].

One of the most concerning resistance mechanisms is the emergence of extended‐spectrum beta‐lactamase (ESBL)–producing Enterobacterales, which is of particular public health concern. Bacteria that produce ESBLs are considered one of the critical priority pathogens by the WHO [7]. Indeed, ESBL‐enzymes hydrolyze most *β*‐lactam antibiotics, including penicillins, cephalosporins, and aztreonam (ATM), but are unable to break down cephamycins or carbapenems [8, 9]. Among livestock, poultry and its products have emerged as critical zoonotic reservoirs of ESBL‐producing Enterobacterales, with *Escherichia coli* and *Klebsiella pneumoniae* being the most common bacteria [10, 11]. The global spread of ESBLs is driven primarily by three gene families *bla*_CTX-M_, *bla*_TEM_, and *bla*_SHV_, which are frequently carried on mobile genetic elements, facilitating horizontal transfer and co‐selection with other resistance determinants [8, 9, 11]. Numerous meta‐analyses report the widespread presence in poultry of ESBL in sub‐Saharan Africa and identify CTX‐M–type enzymes as the primary genetic drivers [12, 13]. However, ESBL colonization levels in poultry vary widely across studies due to differences in detection methods, sample types, geographical locations, production systems, and husbandry practices [13]. Although *E. coli* typically dominates avian ESBL carriage, a study from Zambia reported *Enterobacter* spp. as the dominant genus [14], highlighting important regional variability. This variability underscores the need for context‐specific surveillance.

In Côte d′Ivoire, the poultry sector has expanded rapidly over the past decade due to its role in food security, poverty reduction, and household income generation. However, this growth has been accompanied by widespread unregulated antibiotic use. High‐risk practices, including self‐medication, failure to observe withdrawal periods, and poor biosecurity, have been documented in Grand‐Abidjan farms [15]. These practices have led to antibiotic residues exceeding safety limits in poultry meat and alarmingly high resistance rates to critically important antibiotics such as tetracyclines, sulfonamides, and macrolides in commensal *E. coli* and *Salmonella* from healthy chickens [16, 17].

Despite evidence of ESBL‐producing bacteria in other Ivorian livestock including cattle and rabbits [18, 19], data on ESBL‐producing Enterobacterales in the national poultry reservoir remain strikingly scarce. To address these critical knowledge gaps, we conducted a comparative study on poultry farms in two distinct regions of Côte d′Ivoire: the peri‐urban hub of Grand‐Abidjan and the agropastoral zone of Korhogo. Our primary objectives were to: (i) document antibiotic use practices among poultry farmers; (ii) determine the on‐farm prevalence of ESBL‐producing Enterobacterales in healthy chickens; (iii) identify their AMR profiles; and (iv) the associated genetic determinants of ESBL resistance, focusing on the *bla*_CTX-M_, *bla*_TEM_, and *bla*_SHV_ genes. By generating this essential baseline data, our work is aimed at informing evidence‐based surveillance and antimicrobial stewardship policies within a local One Health framework, helping to mitigate the growing threat of AMR in the Ivorian poultry sector.

## 2. Materials and Methods

### 2.1. Study Approval

This study was approved by the Institutional Ethics Committee of the Institut Pasteur de Côte d′Ivoire (Reference: 07/IPCI/CEIPCI/km). Informed consent was obtained from all participating farmers prior to their inclusion. Participants were informed about the study′s objectives, procedures, voluntary participation, and the right to withdraw at any time. All data were anonymized to ensure confidentiality.

### 2.2. Sampling Area Description

The study was conducted in two geographically distinct regions of Côte d′Ivoire: The Korhogo department (city and surrounding villages) in the northern savanna zone, where mixed crop‐livestock systems dominate, and the peri‐urban expanse of Grand‐Abidjan, surrounding the economic capital (Figure 1). Study sites were selected based on their high density of commercial poultry farms, ensuring representation of key production areas and enabling robust comparative analysis.

**Figure 1:**
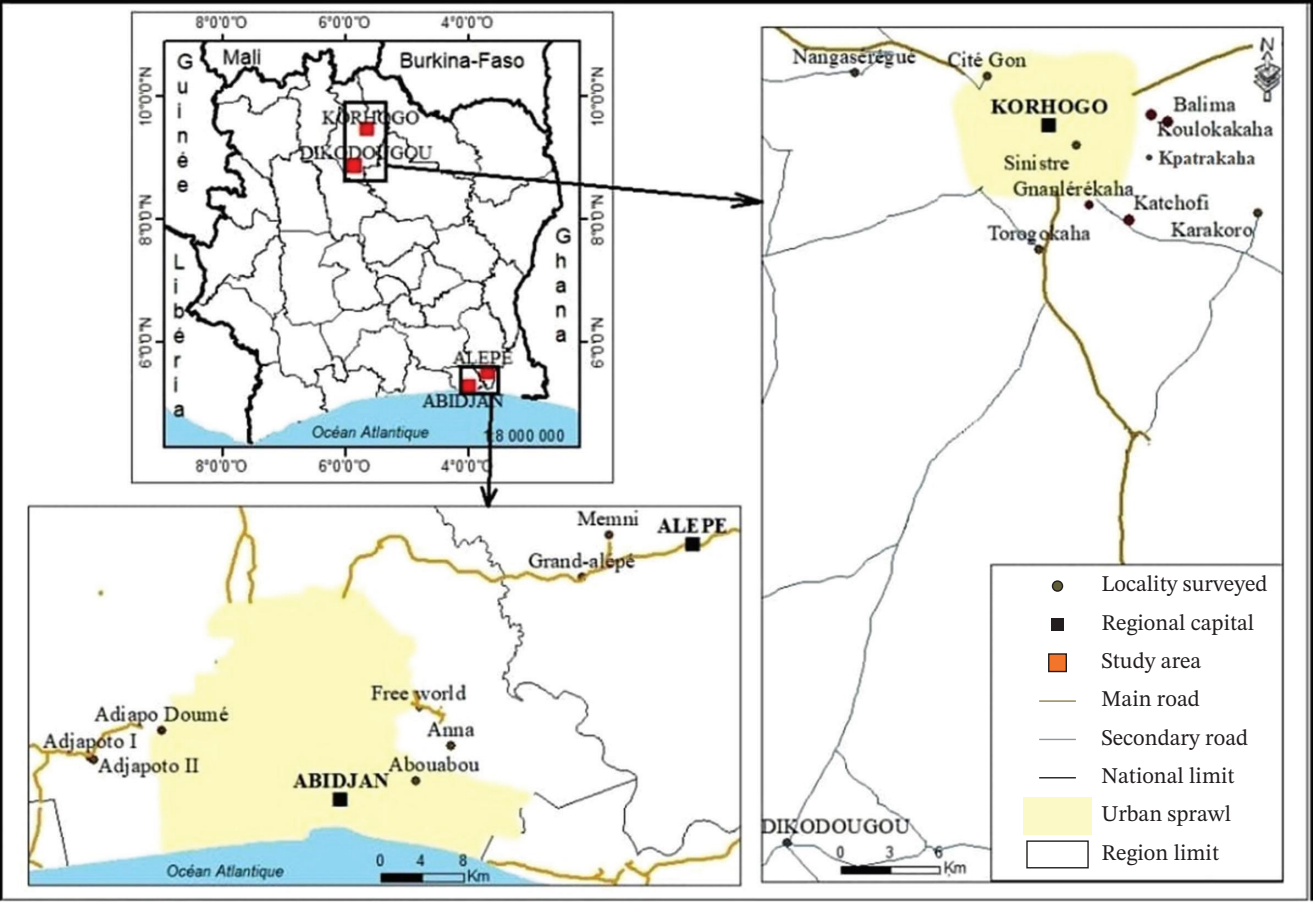
Map of Côte d′Ivoire showing the study area.

### 2.3. Data and Sample Collection

Twenty‐seven poultry farms were randomly selected: 11 in Grand‐Abidjan and 16 in Korhogo. Sampling took place between August and December 2020. Before biological sampling, antibiotic‐use data were collected from 26 poultry farmers who gave informed consent through semi‐structured interviews covering antibiotic types, frequency of use, and withdrawal periods. The remaining farmer consented to fecal sample collection on the farm but declined to participate in the questionnaire survey. For microbiological analysis, fresh fecal samples were collected immediately after defecation from five clinically healthy chickens per farm at distinct locations within each farm, using sterile scoops to reduce environmental contamination. Fecal material from each farm was pooled into a single composite sample and transported to the laboratory at 4°C in an insulated container. Each composite sample was then analyzed using standard microbiological procedures.

### 2.4. Isolation and Identification of Enterobacterales from Poultry Fecal Samples

The isolation and identification of Enterobacterales from the collected samples was achieved through a multi‐step process combining selective enrichment, differential plating, and biochemical profiling. For bacterial enrichment, a 2‐g aliquot of each sample was homogenized in 18 mL of buffered peptone water and incubated at 37°C for 3 h under constant agitation to revive and proliferate the target bacterial populations. Following this initial enrichment, a 100‐*μ*L inoculum from each culture was spread onto MacConkey agar (Bio‐Rad, France) supplemented with ceftazidime (CAZ) at a final concentration of 2 mg/L, thereby imposing a selective pressure for the isolation of presumptive cephalosporin‐resistant strains. Reference strains were used as quality controls: *E. coli* ATCC 25922 served as a negative control to confirm inhibition on CAZ‐supplemented medium, whereas *K. pneumoniae* ATCC 700603 was used as a positive control to verify growth of resistant organisms. Following incubation at 37°C for 24 h, non‐confluent colonies were selected for further purification and preliminary differentiation. Isolates were then subcultured onto Uriselect 4 chromogenic medium (Bio‐Rad, France) to ensure clonal purity and enable preliminary species identification based on colony coloration. After gram staining, presumptive identification was confirmed using conventional biochemical tests including indole production and urease activity according to the manufacturer′s guidelines. For certain isolates, an assessment of key metabolic and enzymatic traits, including gas and hydrogen sulfide (H_2_S) production as well as citrate utilization was performed. Notably, the Uriselect 4 medium enabled early presumptive identification of *E. coli*, and the subsequent indole spot test provided absolute specificity for confirmation of the species [20].

### 2.5. Molecular Identification of *Enterobacter* spp. and *K. pneumoniae*

Following biochemical screening, genomic DNA was extracted from isolates presumptively identified as *Enterobacter* spp. and *K. pneumoniae*. Extraction was performed using the SPIN‐X Viral DNA/RNA (SD Biosensor) Extraction Kit according to the manufacturer′s instructions. All polymerase chain reactions (PCR) were conducted in a Peltier PTC‐200 DNA Engine thermal cycler (Bio‐Rad, United States). For the genus‐level identification of *Enterobacter* spp., a fragment of the *hsp60* gene was amplified using primers *Hsp60-F* (5 ^′^‐GGTAGAAGAAGGCGTGGTTGC‐3 ^′^) and *Hsp60*‐*R* (5 ^′^‐ATGCATTCGGTGGTGATCATCAG‐3 ^′^), following the protocol established by [21]. The thermal cycling conditions comprised an initial denaturation at 95°C for 3 min, followed by 30 cycles of denaturation at 95°C for 15 s, annealing at 55°C for 20 s, and extension at 72°C for 90 s, with a final elongation step at 72°C for 5 min. For the species‐specific confirmation of *K. pneumoniae*, a 108‐bp fragment of the *rpoB* gene was targeted using primers *rpoB-F* (5 ^′^‐CAACGGTGTGGTTACTGACG‐3 ^′^) and *rpoB-R* (5 ^′^‐TCTACGAAGTGGCCGTTTTC‐3 ^′^), as described by [22]. The amplification protocol for this assay included an initial denaturation at 95°C for 15 min, followed by 30 cycles of denaturation at 94°C for 1 min, annealing at 55°C for 1 min, and extension at 72°C for 1 min, concluding with a final extension at 72°C for 10 min. Following amplification, PCR products were resolved by electrophoresis on a 1.5% agarose gel prepared in 1× Tris‐Borate‐EDTA (TBE) buffer. The resulting amplicons were visualized and documented using a gel imaging system (Bioblock Scientific, France), confirming the presence of target DNA fragments of the expected sizes.

### 2.6. Double‐Disc Synergy Test (DDST) and Antimicrobial Susceptibility Testing

Phenotypic confirmation of ESBL production was performed using the DDST following established Kirby–Bauer disk diffusion protocols. Mueller–Hinton agar plates were inoculated with a bacterial suspension adjusted to a 0.5 McFarland standard. An amoxicillin–clavulanic acid disc (AMC, 20/10 *μ*g) was placed at the center, whereas discs of CAZ (10 *μ*g), cefotaxime (CTX, 30 *μ*g), ceftriaxone (CRO, 30 *μ*g), and ATM (30 *μ*g) were positioned 20 mm (center‐to‐center) from the AMC disc. Following incubation at 37°C, a characteristic “keyhole” or distortion of the inhibition zone around any *β*‐lactam disc adjacent to AMC was interpreted as ESBL production [23]. *E. coli* ATCC 25922 and *K. pneumoniae* ATCC 700603 served as negative and positive controls, respectively.

All ESBL‐positive isolates were further tested for susceptibility to a panel of 12 antimicrobial agents using the disk diffusion method on Mueller–Hinton agar. The additional antibiotics tested included: cefoxitin (FOX, 30 *μ*g), cefuroxime (CXM, 30 *μ*g), imipenem (IMP, 10 *μ*g), gentamicin (GMN, 10 *μ*g), ciprofloxacin (5 *μ*g), chloramphenicol (CHL, 30 *μ*g), and trimethoprim–sulfamethoxazole (SXT, 1.25/23.75 *μ*g). Inhibition zone diameters were measured and interpreted according to CA‐SFM/EUCAST guidelines 2023. To avoid clonal bias, only one isolate per specimen exhibiting a given resistance pattern was retained for further analysis.

### 2.7. DNA Extraction and PCR Detection of ESBL‐Encoding Genes

Genomic DNA was extracted from *E. coli* isolates using a thermolysis method. Isolates were cultured overnight on Luria‐Bertani (LB) agar supplemented with 2 mg/L CAZ to ensure selective growth. A few colonies of each isolate were resuspended in 300 *μ*L of nuclease‐free water. The suspension was subjected to a freeze‐thaw lysis procedure involving incubation at −20°C for 15 min, followed immediately by boiling for 15 min. Cellular debris was pelleted by centrifugation at 14,000 rpm for 10 min, and the resulting supernatant, containing the DNA template, was carefully recovered for use in PCR assays. Screening for genes encoding ESBLs was performed using simplex PCR assays targeting *bla*_CTX-M_, *bla*_TEM_, and *bla*_SHV_, following previously described protocols with specific primers and annealing temperature [24] and (Table 1). All isolates exhibiting a phenotypic ESBL profile, including *E. coli*, *Enterobacter* spp., and *K. pneumoniae*, were included in the molecular analysis. Each 20 *μ*L PCR reaction consisted of 5 *μ*L of DNA template, 1X HOT FIREPol Blend Master Mix Ready to Load (Solis BioDyne, Estonia), 0.4 mM of each forward and reverse primer, and nuclease‐free water to reach the final volume. Amplifications were conducted in a Peltier PTC‐200 DNA Engine thermal cycler (Bio‐Rad, United States). For quality control, a clinical ESBL‐producing *K. pneumoniae* strain from the CeDRes collection (University Hospital of Treichville) served as the positive control for *bla*_TEM_ and *bla*_CTX-M_, whereas *K. pneumoniae* ATCC 700603 was used as the positive control for *bla*_SHV_. *E. coli* ATCC 25922 and a no‐template control (nuclease‐free water) were included as negative controls in all PCR runs to monitor for contamination and nonspecific amplification. After amplification, PCR products were resolved by electrophoresis on a 1.5% (w/v) agarose gel run at 130 V for 45 min. Following electrophoresis, the gel was stained with Ethidium Bromide (Promega, United States) for visualization of DNA fragments. The stained amplicons were then visualized under blue light using a gel documentation system.

**Table 1:** Primers used to detect ESBL‐resistance genes.

Target gene	Primers	Sequences (5 → 3)	Annealing (°C)	Size (bp)
*bla*_CTX-M_	CTX‐M‐F	ATG TGC AGY ACC AGT AAR GTK ATG GC	60	593
	CTX‐M‐R	TGG GTR AAR TAR GTS ACC AGA AYS AGC		
*bla*_TEM_	TEM‐F	GCG GAA CCC CTA TTT G	55	964
	TEM‐R	ACC AAT GCT TAA TCA GTG AG		
*bla*_SHV_	SHV‐F	TTA TCT CCC TGT TAG CCA CC	60	795
	SHV‐R	GAT TTG CTG ATT TCG CTC GG		

### 2.8. Data Analysis

Data management and preliminary descriptive analyses were performed using Microsoft Excel. To characterize the dataset, frequency distributions and percentages were calculated for all categorical variables, with 95% confidence intervals reported to indicate the precision of these estimates.

All inferential statistical analyses were performed using R (Version 4.5.0; R Core Team, 2025), with the significance level set at *α* = 0.05. Species occurrence was coded as a binary variable (presence/absence), whereas ESBL gene carriage was treated as a continuous measure. Point‐biserial correlation was therefore suitable for an exploratory assessment of association. The aim was to identify possible species‐specific patterns in ESBL gene distribution, not to establish causality. To assess associations between the primary bacterial species of interest (*E. coli*, *K. pneumoniae*, and *Enterobacter* spp.) and the presence of key *β*‐lactamase‐encoding genes (*bla*_CTX-M_, *bla*_SHV_, *bla*_TEM_, and their combinations), point‐biserial correlation analyses were conducted using the *cor.test* function. To account for multiple comparisons and reduce the risk of Type I error, a more conservative significance level of *α* = 0.05 was applied specifically to these analyses. For each comparison, the correlation coefficient (*r*) and its corresponding *p* value were computed.

## 3. Results

### 3.1. Antimicrobial Use Practices in Poultry Farms

A comprehensive overview of antimicrobial usage practices among consenting poultry farmers in the study area is presented in Table 2, which synthesizes data obtained from 26 of the 27 participating farmers (96.3%) who provided detailed information regarding their veterinary pharmaceutical practices. However, one farmer in Grand‐Abidjan refused to give consent. The analysis revealed that antimicrobial administration was systematically implemented across all surveyed farms, with the therapeutic arsenal predominantly comprising five major classes of antibiotics: tetracyclines (76.9%), macrolides (69.2%), polymyxins (61.5%), aminoglycosides (57.7%), and sulfonamides (53.8%) (Figure S1). Regarding the purposes of antimicrobial application, a majority of farmers (65.38%; 17/26) reported employing these compounds for prophylactic purposes, whereas a subset of four producers, exclusively located in the Korhogo department, acknowledged utilizing antibiotics as growth‐promoting agents. Although all participating farmers reported procuring antibiotics exclusively from licensed veterinary pharmacies, a substantial proportion (34.61%; 9/26) predominantly in the Grand‐Abidjan area admitted acquiring these pharmaceuticals without veterinary prescriptions. The route of antibiotic administration was exclusively through drinking water, with treatment frequencies ranging from weekly to monthly intervals. Notably, the four Korhogo‐based farmers who employed antibiotics for growth promotion administered these compounds on a daily basis. Adherence to recommended treatment protocols was observed in only one‐third of operations (34.61%; 9/26), whereas the majority of farmers (65.39%; 17/26) reported continuing antibiotic administration until clinical symptoms resolution, thereby deviating from established therapeutic guidelines. Furthermore, a concerning practice was identified regarding withdrawal periods, with more than half of the farms (57.7%; 15/26) failing to observe mandatory preslaughter drug cessation intervals prior to marketing poultry products. Finally, an environmental dimension of antimicrobial dissemination was documented through the common practice of drying and subsequently utilizing poultry litter as organic fertilizer, particularly in vegetable farming systems, thereby creating a potential pathway for environmental contamination and antimicrobial dissemination into the food chain.

**Table 2:** Antibiotic practices in poultry farming across the studied farms.

Variable	Details	Frequency		*p*
		Grand‐Abidjan	Korhogo	
		*n*(%)	*n*(%)	
Use antibiotics	Farms using antibiotics	10 (100)	16 (100)	> 0.05
Purpose of antibiotic use	Preventive	7 (70)	10 (62.5)	0.696
	Curative	3 (30)	2 (12.5)	0.271
	Growth promotion	0 (0)	4 (25)	**0.045** ^*^

Source of antibiotics	Veterinary pharmacies	10 (100)	16 (100)	> 0.05
	Purchased without veterinary prescription	6 (60)	3 (18.75)	**0.0315** ^*^

Mode of administration	Drinking water	10 (100)	16 (100)	> 0.05
Treatment frequency	Monthly	6 (60)	8 (50)	0.619
	Weekly	4 (40)	4 (25)	0.420
	Daily	0 (0)	4 (25)	**0.0456** ^*^

Adherence to dosage instructions	Strictly followed	4 (40)	5 (31.25)	0.648
	Extended until disappearance of clinical signs	6 (60)	11 (68.75)	0.648

Adherence to withdrawal periods	Farms not respecting withdrawal periods	5 (50)	10 (62.5)	0.530
Use of poultry droppings	Organic manure	10 (100)	16 (100)	> 0.05

*Note:* Bolded values with an asterisk indicate statistical significance ( ^*^ *p* < 0.05)

### 3.2. Prevalence of ESBL‐Producing Enterobacterales in Poultry Farms

A total of 27 poultry farms were randomly selected, with 16 farms located in the northern savannah region surrounding Korhogo and 11 farms in the southern coastal region of Grand‐Abidjan. Phenotypic screening for cephalosporin resistance, followed by confirmation via the DDST, demonstrated that all cephalosporin‐resistant isolates were indeed ESBL producers (Figure 2A). The contamination of poultry farms with ESBL‐producing Enterobacterales was near‐ubiquitous, with such organisms being detected on 26 of the 27 farms surveyed, resulting in a farm‐level prevalence of 96.3% (95% CI: 81.0–99.9). The single farm that tested negative was situated in the locality of Abouabou, within the Grand‐Abidjan zone (Figure 1).

**Figure 2:**
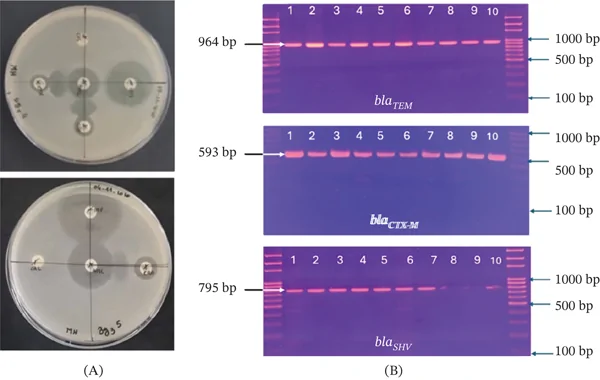
Phenotypic and molecular characterization of ESBL production in poultry Enterobacterales isolates. (A) Phenotypic confirmation by the double‐disc synergy test (DDST) on Mueller–Hinton agar. The enhanced inhibition zones between the centrally placed amoxicillin–clavulanic acid (AMC) disc and the discs of ceftazidime (CAZ) and cefotaxime (CTX) indicate the restoration of cephalosporin activity by the *β*‐lactamase inhibitor, confirming the production of ESBLs. (B) Agarose gel electrophoresis (1.5% w/v) of PCR amplicons specific for ESBL‐encoding genes. From top to bottom: detection of *bla*_TEM_ (964 bp), *bla*_CTX-M_ (593 bp), and *bla*_SHV_ (795 bp) genes. Lane M: 100 bp DNA ladder. Lanes 1–3: *K. pneumoniae* isolates. Lanes 4–7: *Enterobacter* spp. isolates. Lanes 8–10: *Escherichia coli* isolates.

From the positive farms, a total of 90 non‐duplicate ESBL‐producing Enterobacterales isolates were recovered and characterized. The collection comprised 49 isolates from the Grand‐Abidjan region and 41 from Korhogo. Molecular identification, based on species‐specific gene amplification, confirmed all *K. pneumoniae* isolates via the *rpoB* gene (108 bp) and all *Enterobacter* spp. isolates via the *hsp60* gene (341 bp) (Figure S2). The overall species distribution across the entire isolate collection was dominated by *K. pneumoniae* (38/90; 42.2%), followed by *E. coli* (35/90; 38.9%), and *Enterobacter* spp. (17/90; 18.9%).

Marked differences in species distribution were observed between the two agroecological zones. In Grand‐Abidjan, the 49 Enterobacterales isolates were composed of 16 *E. coli*, 16 K*. pneumoniae*, and 17 *Enterobacter* spp. Notably, *Enterobacter* spp. were isolated exclusively from this coastal zone, specifically recovered from farms in the localities of Alépé and Adiopo‐Doumé. In contrast, the species composition in Korhogo was restricted to *E. coli* and *K. pneumoniae*, represented by 19 and 22 isolates, respectively, with a complete absence of *Enterobacter* spp.

### 3.3. Distribution of ESBL Genes in Enterobacterales Isolates

All 90 phenotypically confirmed ESBL‐producing Enterobacterales carried at least one of the three target genes *bla*_CTX-M_, *bla*_SHV_, *bla*_TEM_ (Figure 2B). The overall distribution showed a predominance of *bla*_CTX-M_ (77.8%, 70/90), followed by *bla*_SHV_ (54.4%, 49/90) and *bla*_TEM_ (31.1%, 28/90). The complete distribution of these resistance determinants is presented in Table 3.

**Table 3:** Genetic profile of ESBL‐type genes among Enterobacterales isolates.

Pattern of genes	*E. coli*	*Enterobacter* spp.	*K. pneumoniae*	Total
	*n*(%)	*n*(%)	*n*(%)	
*bla*_CTX-M_	21 (60)	2 (11.76)	5 (13.16)	28 (31.11)
*bla*_SHV_	8 (22.86)	3 (17.65)	6 (15.79)	17 (18.89)
*bla*_TEM_ *+ bla*_CTX-M_	6 (17.14)	4 (23.53)	3 (7.89)	13 (14.44)
*bla*_SHV_ *+ bla*_CTX-M_	0 (0)	5 (29.41)	12 (31.58)	17 (18.89)
*bla*_TEM_ *+ bla*_SHV_	0 (0)	0 (0)	3 (7.89)	3 (3.33)
*bla*_TEM_ *+ bla*_SHV_ *+ bla*_CTX-M_	0 (0)	3 (17.65)	9 (23.68)	12 (13.33)
Total	35 (38.89)	17 (18.89)	38 (42.22)	90 (100)

Species‐specific analysis revealed distinct genotypic profiles. Among *E. coli* isolates (*n* = 35), *bla*_CTX-M_ was overwhelmingly predominant, identified in 77.1% (27/35) of strains, whereas bla_SHV_ was considerably less prevalent (8/35, 22.9%). The concurrent carriage of two genes was observed in a minority of *E. coli* isolates, with only six (17.1%) co‐harboring both *bla*_CTX-M_ and *bla*_TEM_. In contrast, *K. pneumoniae* (*n* = 38) exhibited a high prevalence of both *bla*_CTX-M_ (29/38, 76.3%) and *bla*_SHV_ (30/38, 78.9%), whereas *bla*_TEM_ was detected in 39.5% (15/38) of these isolates. A significant proportion of *K. pneumoniae* strains harbored multiple ESBL genes, with 71% (27/38) carrying more than one determinant. For *Enterobacter* spp. (*n* = 17), *bla*_CTX-M_ remained the most prevalent gene (14/17, 82.3%), followed by *bla*_SHV_ (11/17, 64.7%) and *bla*_TEM_ (7/17, 41.1%). Similar to *K. pneumoniae*, the carriage of multiple ESBL genes was a frequent occurrence in this genus, observed in 71% (12/17) of isolates.

Overall, precisely half of the isolates in the entire collection (45/90, 50%) were found to carry two or all three of the ESBL genes investigated. This pattern of multigene carriage was notably more common in *K. pneumoniae* and *Enterobacter* spp. than in *E. coli*. The most prevalent combination among these multiresistant isolates was *bla*_CTX-M_ + *bla*_SHV_, a profile identified in 31.6% of *K. pneumoniae* and 29.4% of *Enterobacter* spp. isolates. A particularly striking observation was the complete absence of *bla*_TEM_ as a solitary ESBL gene; when present, it was detected exclusively in combination with either *bla*_CTX-M_, *bla*_SHV_, or both.

### 3.4. Correlation Analysis of Bacteria Species and ESBL Genes

Pairwise correlation analysis revealed species‐specific patterns of association between bacterial species and ESBL resistance genes (Figure 3). For *K. pneumoniae*, a moderate positive correlation was observed with *bla*_SHV_ (*r* = 0.42, *p* < 0.01), together with weaker but significant correlations with the *bla*_CTX-M_/*bla*_SHV_ combination (*r* = 0.28, *p* < 0.05) and the triple combination *bla*_CTX-M_/*bla*_SHV_/*bla*_TEM_ (*r* = 0.26, *p* < 0.05). In contrast, *E. coli* was negatively correlated with *bla*_SHV_ (*r* = −0.51, *p* < 0.001), *bla*_TEM_ (*r* = −0.24, *p* < 0.05), and several gene combinations (*r* = −0.38 to − 0.31, *p* < 0.05). These findings suggest that the distribution of ESBL genes may differ between bacterial species, with certain genes or gene combinations occurring more frequently in some species than in others. No significant correlations were observed for *Enterobacter* spp. (*r* = 0.05 to 0.13; *p* > 0.05 for all comparisons).

**Figure 3:**
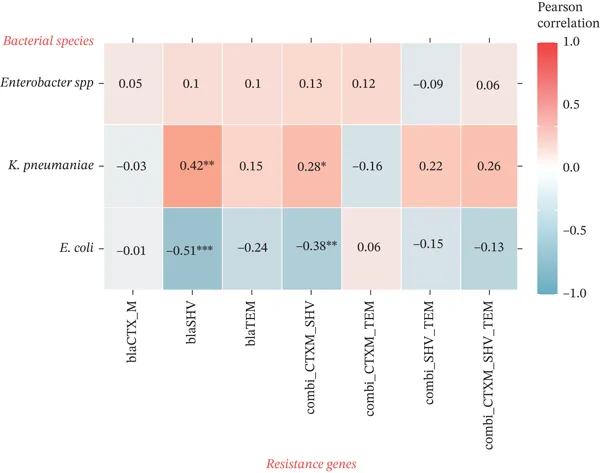
Pearson correlation matrix (*r* values) bacterial species with resistance genes. Statistically significant correlation results indicated by asterisks and threshold for correlation strength classifications. −0.24 ≤ *r* ≤ 0.31 (*p* < 0.05; statistically significant correlation); −0.38 ≤ *r* ≤ 0.42 (*p* < 0.01; highly significant correlation); *r* = 0.51 (∗∗*p* < 0.001; statistically very strong correlation).

### 3.5. AMR Profiles of ESBL‐Producing Isolates

The complete antimicrobial susceptibility profiles for *E. coli*, *Enterobacter* spp., and *K. pneumoniae* are detailed in Table 4. As expected from phenotypic and genotypic confirmation, all isolates demonstrated 100% resistance to third‐generation cephalosporins (CAZ, CTX, and CRO) and the monobactam ATM.

**Table 4:** Prevalence of antibiotic resistance profiles of Enterobacterales isolates.

Antibiotics	*E. coli*		*Enterobacter* spp.		*K. pneumoniae*		Total	
	(*N* = 35)		(*N* = 17)		(*N* = 38)			
	*n*	(%)	*n*	(%)	*n*	(%)	*n*	(%)
AMC	18	51.4	11	64.7	23	60.5	52	57.78
CXM	35	100	17	100	38	100	90	100
FOX	2	5.7	17	100	0	0	19	21.11
CRO	35	100	17	100	38	100	90	100
CTX	35	100	17	100	38	100	90	100
CAZ	35	100	17	100	38	100	90	100
ATM	35	100	17	100	38	100	90	100
IMP	0	0	0	0	0	0	0	0
GEN	11	31.4	1	5.9	14	36.8	26	28.89
CIP	8	22.9	9	52.9	23	60.5	40	44.44
SXT	23	65.7	17	100	27	71.1	67	74.44
CHL	2	5.7	1	5.9	3	7.9	6	6.67
MDR	12	32.3	9	52.9	24	63.1	45	50

Abbreviations: AMC, amoxicillin–clavulanic acid; ATM, aztreonam; CAZ, ceftazidime; CHL, chloramphenicol; CIP, ciprofloxacin; CRO, ceftriaxone; CTX, cefotaxime; CXM, cefuroxime; FOX, cefoxitin; GEN, gentamicin; IPM, imipenem; STX, trimethoprim/sulfamethoxazole.

Notable interspecies differences in susceptibility to other antibiotic classes were observed. *Enterobacter* spp. isolates were characterized by entire resistance to FOX (100%), in contrast to the susceptible profiles of *E. coli* and *K. pneumoniae*. This genus also exhibited the highest co‐resistance rates, notably to trimethoprim–sulfamethoxazole (100%) and AMC (64.7%). Resistance to ciprofloxacin was most prevalent in *K. pneumoniae* (60.5%) and *Enterobacter* spp. (52.9%), whereas GEN resistance was highest in *K. pneumoniae* (36.8%) and E.coli (31.4%). Critically, all isolates remained susceptible to the carbapenem antibiotic, IMP.

Multidrug resistance (MDR) was widespread, identified in 50% of all isolates. The prevalence of MDR was highest among *K. pneumoniae*, affecting 63% of isolates within this species. Furthermore, the distribution of ESBL‐encoding genes, analyzed in conjunction with the antibiotic resistance profiles and geographic origins (Grand‐Abidjan or Korhogo) of the Enterobacterales isolates, is presented in Figure 4. This analysis revealed that isolates from both geographic locations exhibited broadly similar characteristics.

**Figure 4:**
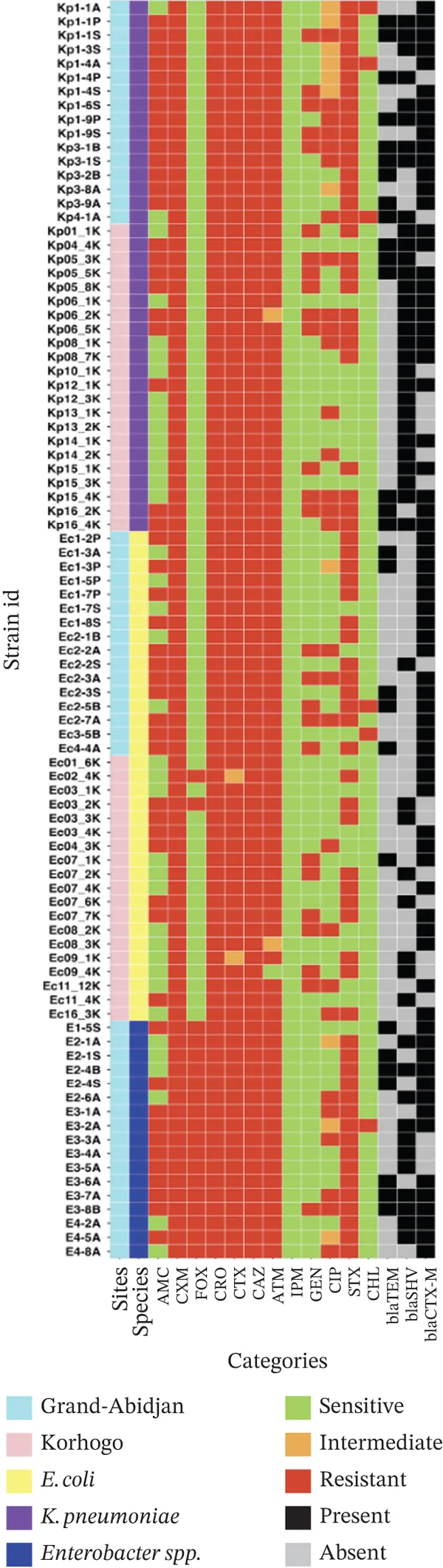
Hierarchical clustering analysis of phenotypic resistance and associated extended‐spectrum *β*‐lactamase (ESBL) gene carriage in poultry‐derived Enterobacterales isolates. The heatmap illustrates co‐occurrence patterns between antibiotic susceptibility profiles (phenotype) and the presence of ESBL genes (*bla*_CTX-M_, *bla*_SHV_, and *bla*_TEM_) across *E. coli*, *K. pneumoniae*, and *Enterobacter* spp. isolates. Hierarchical clustering (rows) groups isolates with similar resistance and genetic profiles, whereas clustering of antibiotics and genes (columns) reveals associations between specific resistance mechanisms and drug classes. Isolate origin (farm/location; Grand‐Abidjan and Korhogo) is annotated to visualize potential epidemiological clustering.

## 4. Discussion

The global rise of ESBL‐producing Enterobacterales in food animals has become a pressing public health concern, and our investigation into poultry farms in Côte d′Ivoire reveals an alarming prevalence of these resistant organisms. An isolate was considered an ESBL producer when phenotypic and molecular results converged. Phenotypic testing showed resistance to third‐generation cephalosporins and ATM in all isolates; adding clavulanic acid restored susceptibility and yielded the characteristic champagne–cork synergy image on double‐disc assays [23]. This pattern was mirrored molecularly, with each isolate harboring at least one of the principal ESBL determinants, that is, *bla*_CTX-M_, *bla*_SHV_, or *bla*_TEM_. We did not test for other ESBL families, such as *bla*_OXA_, *bla*_PER_, *bla*_VEB_, or *bla*_GES_ [9]. Still, the methods used align with Drieux et al.′s approach for detecting ESBL‐producing isolates [23]. Taken together, the data support classifying all study isolates as ESBL producers. Variant‐level confirmation within the *bla*_CTX-M_, *bla*_SHV_, and *bla*_TEM_ families would, however, need sequencing.

Strikingly, 96.3% of the farms sampled were positive, indicating that ESBL‐producing Enterobacterales colonization is not sporadic but deeply entrenched within the production system. This finding aligns with and amplifies evidence of ESBL spread across livestock sector. Indeed, previous national studies have reported these resistant bacteria in cattle: 52.9% [18] and recently, in rabbits: 2.36% [19]. When viewed together, this work shows the widespread dissemination of ESBL‐producing Enterobacterales in Ivorian livestock. Notably, the near‐ubiquitous prevalence observed in poultry significantly exceeds rates reported for other domestic animals, solidifying its status as a primary reservoir of ESBL‐producing Enterobacterales [11, 25, 26]. Consequently, the implications for human health are direct and serious. The dominance of ESBL‐producing Enterobacterales in poultry, one of the major food sources, creates a potent conduit for zoonotic transmission [10, 13, 27, 28].

The colonization rate of ESBL‐producing Enterobacterales observed in this study is among the highest reported in the poultry sector in African countries. For instance, studies have reported rates of 25% in Nigeria [29], 13.6% in Zambia [14], 8.4% in Ethiopia [30], and 13.2% in Kenya [31]. These marked disparities likely reflect underlying methodological, epidemiological, and ecological differences, including variations in livestock husbandry practices, local antimicrobial pressure, and specific screening protocols [13]. In our investigation, direct culture on MacConkey agar supplemented with 2 mg/L CAZ proved to be a highly sensitive and specific approach for detecting ESBL producers. Notably, every preselected isolate was subsequently confirmed both phenotypically and genotypically as an ESBL producer, underscoring the robustness of our screening protocol. This use of CAZ supplementation aligns with the findings of Giriyapur et al. [32], who similarly demonstrated its excellent performance as an early screening agent for ESBL‐producing bacteria, reporting high specificity and sensitivity.

Our analysis of ESBL‐producing Enterobacterales in poultry revealed a distinct taxonomic profile, with *K. pneumoniae* (42.2%) and *E. coli* (38.9%) as the predominant species, followed by *Enterobacter* spp. (18.9%). This distribution contrasts with numerous studies across poultry farming systems in developing countries, where *E. coli* is consistently reported as the dominant ESBL‐producing species [25, 29–31, 33, 34]. Nevertheless, our findings align with the recognized clinical significance of these pathogens, which are leading causes of ESBL‐associated infections in humans across Africa [35]. The preeminence of *K. pneumoniae* and *E. coli* is consistent with their established role as primary carriers of ESBL genes globally highlighting their central position within the One Health paradigm where animal and human health intersect [11].

Notably, the prevalence of ESBL‐producing *E. coli* observed in our study (38.9%) was higher than that reported in Zambia; 20.1% [36], comparable with findings from Nigeria; 37.8% [37], yet lower than those documented in Ghana; 56.2% [38] and Senegal, 61.7% [39]. Characterization of resistance determinants revealed a predominance of the *bla*_CTX-M_ gene in *E. coli* isolates, frequently alone or occasionally in combination with *bla*_TEM_. This genetic profile aligns with global findings and reinforces the pivotal role of CTX‐M‐type enzymes in driving the dissemination of ESBL‐producing *E. coli* across the region [12, 13].

A striking finding was the emergence of *K. pneumoniae* as the most frequently isolated species. This is a serious public health concern given the pathogen′s inherent virulence and proficiency in resistance gene transmission [40, 41]. Molecular profiling revealed a complex resistance background. Most *K. pneumoniae* isolates harbored *bla*_SHV_ and *bla*_CTX-M_ genes, with a substantial majority (71%) co‐expressing these *β*‐lactamase genes along with *bla*_TEM_. This resistance pattern closely aligns with profiles reported in clinical isolates from Côte d′Ivoire [42] and in environmental samples from Burkina Faso, a neighboring country [43]. Notably, the *bla*_SHV_ gene was positively correlated with *K. pneumoniae*, a finding consistent with studies in the poultry sector in Bangladesh and within a One Health framework in China [44, 45]. This frequent association reflects the well‐documented role of *K. pneumoniae* as a major reservoir for *bla*_SHV_ [9]. Together, these findings point to a concerning epidemiological link, suggesting a shared reservoir and continuous exchange of resistant strains across animal, human, and environmental compartments.

Among the identified Enterobacterales, *Enterobacter* spp. was the least represented, with a frequency of 18.9%, and was isolated exclusively from farms in Grand‐Abidjan. This proportion is higher than the 14% reported in Zambia from diseased broiler chickens [14]. Its notable occurrence in this region suggests a potential emergence within the Ivorian animal reservoir, particularly in the Grand‐Abidjan area. A key genetic characteristic of the isolated *Enterobacter* spp. was a strong propensity for co‐expression of ESBL genes, observed in nearly 71% of strains. This profile mirrors a common trait of *K. pneumoniae*, pointing to an active dynamic of horizontal gene exchange between these two pathogenic species within Ivorian poultry farms. This complex resistance landscape stands in contrast to reports from Zambia, where only the *bla*_TEM_ gene has been reported in avian isolates [14], underscoring the distinct diversity and evolution of resistance mechanisms across the continent.

Phenotypically, the bacterial isolates exhibited a concerning resistance profile. Beyond expected *β*‐lactam resistance, high rates were observed for sulfamethoxazole–trimethoprime (75%) and ciprofloxacin (45%), levels comparable with those reported in Senegal and Nigeria [37, 39]. However, resistance to the AMC combination (57%) stands out as particularly elevated in Côte d′Ivoire, contrasting sharply with the 0% reported in those same regional studies [37, 39]. This disparity indicates intense local selective pressure, potentially linked to the production of other beta‐lactamases, which are able to reduce affinity for lactamase inhibitors such as clavulanate [8, 9], a mechanism that compromises this essential therapeutic option.

Concurrently, only half (50%) of the isolates exhibited a MDR phenotype, with the highest prevalence observed in *K. pneumoniae* (63%) and *Enterobacter* spp. (52%), compared with 32% in *E. coli*. This overall MDR frequency is lower than those reported in numerous studies across the region, where rates frequently reach up to 80% [17, 30, 37–39]. This prevalence is likely underestimated, as our test panel did not include antibiotic classes heavily used on farms. Indeed, our practice analysis revealed the common, nonregulated use of five classes of antibiotic including polymyxins (colistin), tetracyclines, macrolides, aminoglycosides, and sulfonamides with tetracyclines occupying a predominant role as consistently report across Africa countries [13, 46]. The spread of ESBL‐producing Enterobacterales could be partly attributable to coselection, as hypothesized by Cassella et al. [47]. Indeed, plasmids harboring ESBL genes often also carry resistance determinants for other antibiotic classes (e.g., aminoglycosides, sulfonamides, quinolones, colistin, and tetracyclines). As a result, the misuse of these non–*β*‐lactam agents may inadvertently select for the entire plasmid, thereby promoting the persistence and dissemination of ESBL‐producing strains even in the absence of direct cephalosporin pressure.

One of the more reassuring findings of this study was that every ESBL‐producing Enterobacterales isolate remained susceptible to IMP, a carbapenem reserved for last‐line treatment. This agrees with earlier reports from Senegal and Ghana, where carbapenem resistance has remained uncommon among poultry‐associated Enterobacterales [38, 39]. Nevertheless, this result should not be read as grounds for complacency. The frequent and often unregulated use of critically important antibiotics in Ivorian poultry farming, as documented here, creates conditions that could favor the emergence of carbapenem resistance in the future. This pattern also underscores the need for surveillance that anticipates resistance rather than simply documenting it. Sustaining the efficacy of carbapenems in both human and veterinary medicine will therefore require a coordinated One Health approach, one that combines stronger surveillance, prudent antimicrobial use policies, and improved infection prevention and control in livestock production systems.

In the present study, high‐risk practices have been reported, including widespread self‐medication, prophylactic and growth promoter antibiotic use and poor biosecurity, as previously documented among poultry farmers in sub‐Saharan Africa [46]. In addition, the routine use of antimicrobials classified by the WHO as critically important for human medicine raises profound public health concerns. Notably, macrolides and polymyxins including colistin, a last‐resort antibiotic for human medicine are employed with frequency in poultry production (61%), despite being strictly regulated in the European Union and multiple Asian countries in food animal production [48]. Furthermore, over half of surveyed farmers (55.6%) disregard mandatory withdrawal periods before slaughter, which may lead to detectable antibiotic residues in meat that surpass safe limits, as revealed in Daloa [16]. Equally concerning is the widespread, unregulated use of poultry litter as crop fertilizer, which may facilitate the environmental spread of ESBL genes into soils, produce, and water resources creating pathways for transmission to humans through food and water [49, 50]. These findings highlight an urgent need for a coherent, locally adapted One Health strategy. Effective containment of ESBL‐producing Enterobacterales at the human–animal–environment interface will require integrated efforts: enhanced resistance surveillance, stringent controls on veterinary antibiotic access, and targeted training for poultry value‐chain actors.

## 5. Conclusion

This study shows that poultry farms in Côte d′Ivoire are major reservoirs of ESBL‐producing Enterobacterales and highlights farming practices that promote the emergence of MDR bacteria. Molecular analysis revealed species‐specific resistance profiles: *K. pneumoniae* and *Enterobacter* spp. frequently harbored multiple ESBL genes, whereas *E. coli* isolates mainly carried *bla*_CTX-M_ as the sole ESBL determinant. Future work aimed at clarifying how dissemination differs by species would benefit from plasmid replicon typing, conjugation assays, and more complete plasmid characterization. These findings also strengthen the case for a One Health approach that tracks resistance gene transmission across human, animal, and environmental settings. Pairing such an approach with high‐resolution molecular typing, notably multilocus sequence typing (MLST) and whole‐genome sequencing, would generate the evidence needed to guide surveillance priorities and antimicrobial stewardship strategies suited to the Ivorian context.

## Author Contributions

Christiane You Essoh: conceptualization, methodology, data curation, investigation, validation, formal analysis, project administration, resources, writing—original draft, and writing—review and editing. Gilles Alex Pakora: conceptualization, methodology, data curation, validation, resources, writing—original draft, and writing—review and editing. Daouda Toure: methodology, validation, formal analysis, and writing—review and editing. Inès Cinthia A. Yebouet: investigation, formal analysis, and writing—review and editing. Bérenger Aristide A. Ako: conceptualization, methodology, validation, formal analysis, visualization, and writing—review and editing. Jemima Arlette Ayebie and Naomie Dali: investigation and writing—review and editing. Sinon‐Pierre Assanvo N′guetta and Adèle Kakou‐N′douba: methodology, validation, supervision, resources, writing—original draft, and writing—review and editing.

## Funding

No funding was received for this manuscript.

## Ethics Statement

This study was approved by the Institutional Ethics Committee of the Institut Pasteur of Côte d′Ivoire (Reference: 07/IPCI/CEIPCI/km).

## Conflicts of Interest

The authors declare no conflicts of interest.

## Supporting information


**Supporting Information** Additional supporting information can be found online in the Supporting Information section. Figure S1: Proportion of poultry farmers using different classes of antibiotics. Figure S2: Agarose gel electrophoresis of species‐specific PCR amplicons for *Enterobacter* spp. (*hsp60*, 341 bp) and *Klebsiella pneumoniae* (*rpoB*, 108 bp) with lane M: 100 bp DNA ladder and Lanes 1–10: representative positive isolates.

## Data Availability

The data that support the findings of this study are available from the corresponding author upon reasonable request.
