# Supplementary material for: Antimicrobial Usage and Presence of Extended‐Spectrum β‐Lactamase–Producing Enterobacterales in Healthy Chickens From Farms in Grand‐Abidjan and Korhogo, Côte d′Ivoire
Source: Int J Microbiol. 2026 Oct 21;2026:7418124. doi: 10.1155/ijm/7418124 (PMC13685654; doi:10.1155/ijm/7418124)
Supplement: Supplementary file 1 [file IJM-2026-7418124-s001.docx]

**Supplementary Materials**

**Supplementary Figure 1**. Proportion of poultry farmers using different classes of antibiotics

**Supplementary Figure 2**. Agarose gel electrophoresis of species-specific PCR amplicons for Enterobacter spp. (hsp60, 341 bp) and Klebsiella pneumoniae (rpoB, 108 bp) with lane M: 100 bp DNA ladder and lanes 1–10: representative positive isolates.
